# Comparison of Different Systemic Therapeutic Regimes in Resectable Soft-Tissue Sarcoma—Results of a Network Meta-Analysis

**DOI:** 10.3390/cancers13225631

**Published:** 2021-11-11

**Authors:** Jan Haussmann, Christiane Matuschek, Edwin Bölke, Balint Tamaskovics, Stefanie Corradini, Rüdiger Wessalowski, Kitti Maas, Livia Schmidt, Klaus Orth, Matthias Peiper, Verena Keitel, Torsten Feldt, Björn-Erik Ole Jensen, Tom Luedde, Johannes Fischer, Wolfram Trudo Knoefel, Hany Ashmawy, Alessia Pedotoa, Kai Kammers, Wilfried Budach

**Affiliations:** 1 Department of Radiation Oncology, Heinrich Heine University, 40225 Dusseldorf, Germany; jan.haussmann@med.uni-duesseldorf.de (J.H.); matuschek@med.uni-duesseldorf.de (C.M.); Balint.tamaskovics@med.uni-duesseldorf.de (B.T.); kitti.maas@med.uni-duesseldorf.de (K.M.); Livia.Schmidt@med.uni-duesseldorf.de (L.S.); klausorth@gmx.de (K.O.); peiper@mail.de (M.P.); Wilfried.Budach@med.uni-duesseldorf.de (W.B.); 2 Department of Radiation Oncology, University Hospital LMU University, 81377 Munich, Germany; Stefanie.Corradini@med.uni-muenchen.de; 3 Department of Paediatric Haematology and Oncology, Medical Faculty, Heinrich-Heine-University Dusseldorf, 40225 Dusseldorf, Germany; wessalowski@med.uni-duesseldorf.de; 4 Department of Gastroenterology, Hepatology and Infectious Diseases University Hospital Dusseldorf, Medical Faculty, Heinrich-Heine-University Dusseldorf, Moorenstr. 5, 40225 Dusseldorf, Germany; keitelan@hhu.de (V.K.); torsten.feldt@med.uni-duesseldorf.de (T.F.); Bjoern-ErikOle.Jensen@med.uni-duesseldorf.de (B.-E.O.J.); tom.luedde@med.uni-duesseldorf.de (T.L.); 5 Institute for Transplant Diagnostics and Cell Therapeutics, University Hospital Dusseldorf, Medical Faculty, Heinrich-Heine-University, 40225 Dusseldorf, Germany; jfischer@med.uni-duesseldorf.de; 6 Department of Surgery and Interdisciplinary Surgical Intensive Care Unit, Medical Faculty, Heinrich Heine University, 40225 Dusseldorf, Germany; WolframTrudo.Knoefel@med.uni-duesseldorf.de (W.T.K.); Hany.Ashmawy@med.uni-duesseldorf.de (H.A.); 7 Department of Anesthesiology, Memorial Sloan Kettering Cancer Center, New York, NY 10065, USA; pedotoa@mskcc.org; 8 Division of Biostatistics and Bioinformatics, Department of Oncology, The Sidney Kimmel Comprehensive Cancer Center at Johns Hopkins, The Johns Hopkins University School of Medicine, Baltimore, MD 21205, USA; kai.kammers@jhu.edu

**Keywords:** network meta-analysis, chemotherapy, hyperthermia, surgery, overall survival

## Abstract

**Simple Summary:**

This analysis was conducted to compare different systemic treatment options in the therapy of localized and resectable soft-tissue sarcoma. We tested these in a network against the current standard of resection with or without radiotherapy, which allowed direct and indirect comparisons. Chemotherapy given after the local therapy and chemotherapy added to regional hyperthermia before the operation were associated with a longer survival and likeliness of being disease free and alive. Chemotherapy tailored to the specific subtype of sarcoma did not improve outcomes. Adjuvant chemotherapy might be more helpful in male rather than female patients.

**Abstract:**

Background: The standard treatment of high-risk soft-tissue sarcoma consists of surgical resection followed by risk-adapted radiation therapy. Further treatment options that may improve local and systemic tumor control, including chemotherapy, are not well established. Due to the heterogeneity of the disease, different systemic approaches as well as their application at different time points have been attempted. Methods: We conducted a systematic literature search for randomized clinical trials in the treatment of localized, resectable high-risk adult soft-tissue sarcoma comparing different treatment modalities according to the PRISMA guidelines. We extracted published hazard ratios and number of events for the endpoints overall and disease-free survival (OS; DFS) as well as local and distant recurrence-free interval (LRFI; DRFI). The different modalities were compared in a network meta-analysis against the defined standard treatment surgery ± radiotherapy using the inverse-variance heterogeneity model. Results: The literature search identified 25 trials including 3453 patients. Five different treatment modalities were compared in the network meta-analysis. The addition of adjuvant chemotherapy significantly improved OS compared to surgery ± radiotherapy alone (HR = 0.86; CI-95%: 0.75–0.97; *p* = 0.017). Likewise, neoadjuvant chemotherapy combined with regional hyperthermia (naCTx + HTx) also led to superior OS (HR = 0.45; CI-95%: 0.20–1.00; *p* = 0.049). Both neoadjuvant chemotherapy alone (naCTx) and perioperative chemotherapy (periCTx) did not improve OS (HR = 0.61; CI-95%: 0.29–1.29; *p* = 0.195 and HR = 0.66; CI-95%: 0.30–1.48; *p* = 0.317, respectively). Histology-tailored chemotherapy (htCTx) also did not improve survival compared to surgery ± radiotherapy (HR = 1.08; CI-95%: 0.45–2.61; *p* = 0.868). The network analysis of DFS, LRFI, and DRFI revealed a similar pattern between the different treatment regimens. Adjuvant chemotherapy significantly improved DFS, LRFI, and DRFI compared to surgery ± radiotherapy. In direct comparison, this advantage of adjuvant chemotherapy was restricted to male patients (HR = 0.78; CI-95%: 0.65–0.92; *p* = 0.004) with no effect for female patients (HR = 1.08; CI-95%: 0.90–1.29; *p* = 0.410). Conclusions: Standardized chemotherapy in high-risk soft-tissue sarcoma appears to be of added value irrespective of timing. The benefit of adjuvant chemotherapy seems to be restricted to male patients. The addition of regional hyperthermia to neodjuvant chemotherapy achieved the best effect sizes and might warrant further investigation.

## 1. Background

Limb-sparing surgery with adjuvant or neoadjuvant radiotherapy has been accepted as the standard treatment for resectable soft-tissue sarcomas located in the extremities, trunk, or head and neck region [1,2,3]. The addition of radiotherapy may eliminate the need for amputation and leads to a significant improvement in local control rates in both low- and high-grade sarcomas. For patients with retroperitoneal sarcoma the value of radiotherapy has not been universally established [4]. However, sarcomas with deep muscle invasion, large tumor size or higher tumor grading particularly have a significant risk of distant disease burden metastasizing predominantly to the lungs and, also less frequently, to the liver and bones [5]. Historically, these findings led to several attempts in improving systemic control by using different chemotherapy regimes in addition to local therapies in high-risk sarcomas. A pooled analysis of several randomized trials on individual patient data detected a small benefit in overall survival achieved by improving local rates as well as distant control by the addition of chemotherapy compared to local therapy alone [6]. A subsequent updated analysis revealed a comparable increase in overall survival by the addition of combined doxorubicin- and ifosfamide-based chemotherapy [7]. However, a subsequent large multicenter study by the European Organization for Research and Treatment of Cancer (EORTC) was unable to replicate this improvement in overall survival using adjuvant chemotherapy with doxorubicin and ifosfamide [8]. One of the explanations for the relatively small benefit of postoperative chemotherapy in this EORTC-study could be incomplete dosing and application of chemotherapy due to postoperative complications which may result in significant treatment delay. For this reason, several attempts have been made during the last decade to shift chemotherapy to the neoadjuvant or perioperative setting as known from other cancer entities [9]. In addition, combinations with hyperthermia have also been tested showing promising results [10].

Overall, soft-tissue sarcomas of the extremities and trunk are a very heterogeneous group of tumors consisting of many differently behaving histologically types with varying potential for local and distant recurrence. This is the rationale for a tumor-specific chemotherapeutic approach, consisting of a tailored systemic therapy for each subtype of sarcoma. At present, there is little knowledge about the optimal treatment regimen and sequencing to achieve the best outcome. Therefore, we sought to analyze the different approaches in a network meta-analysis.

## 2. Methods

We conducted a literature search in the electronic databases MEDLINE and EMBASE until June 2021, without restrictions regarding language or publication status, for controlled randomized trials including surgery with or without radiation therapy and different timings or different therapeutic strategies of systemic therapy in resectable soft-tissue sarcoma in accordance with the PRISMA guidelines for network meta-analyses [11,12]. The chosen key words were “soft-tissue sarcoma” AND “chemotherapy” AND “localized” with a restriction for type of articles to clinical trials only. This meta-analysis is registered in PROSPERO (ID: 289154).

We excluded trials comparing different chemotherapy regimens in the same oncological setting, e.g., regimen 1 vs. regimen 2 in the adjuvant setting, as the comparison of different systemic substances and dosages was not subject of this analysis. Patients not amendable for local therapy due to locally advanced disease or metastatic spread were also excluded from the analysis. Studies including GIST or any pediatric soft-tissue sarcoma were not part of this analysis. We extracted the hazard ratios or event numbers from the published randomized trials. In studies where only survival curves were available, we estimated the corresponding hazard ratios using the method published by Parmar et al. and Tierney et al. [13,14].

The quantitative meta-analysis of data from individual patients of the ‘Sarcoma Meta-analysis Collaboration’ group provided the best estimates of effect sizes for the analyzed endpoints [6]. Unfortunately, further follow-ups of the included studies were not available for analyzation yet. Therefore, we included the endpoints from this publication and added other studies only to our network analyses if they reported matching endpoints.

A meta-analysis of effect sizes on overall survival (OS), disease-free survival (DFS), local recurrence-free interval (LRFI), and distant relapse-free interval (DRFI) was performed using the inverse variance heterogeneity model (ivhet). The definition of the analyzed endpoints was derived from the published trials. A correction of the follow-up time in the LRFI and DRFI odds ratios was not possible due to the small number of studies and different median follow-up times. All statistical analyses were carried out using the Microsoft Excel (Microsoft, Redmond, WA, USA) add-in MetaXL (version 5.3, School of Public Health, University of Queensland, Brisbane, Australia). Due to possible heterogeneity of the study populations, the inverse variances of heterogeneity model (ivhet) by Doi et al. were chosen as a comparison method [15]. This method favors larger trials, uses a more conservative estimate of the confidence limits, and produces fewer observed variances compared to the random effects model. Zero-event correction was applied where appropriate. For the analysis of heterogeneity or inconsistency in the network, we calculated the weighted average H over all comparisons. Values below 3 were considered as an indication of minimal inconsistency.

A subgroup analysis of matching endpoints and cohorts was possible for the direct comparison of surgery ± radiotherapy versus surgery ± radiotherapy + adjuvant chemotherapy. The effect of adjuvant chemotherapy with regard to gender of 2 EORTC trials was available from one trial only [16,17] and as cumulative effect from both trials. Both results were included, however, the weight of the effect size in the cumulative publication was reduced according to smaller sample size of the smaller trial [8]. Statistical significance level was set at *p*-values lower than 0.05.

## 3. Results

**Figure 1:**
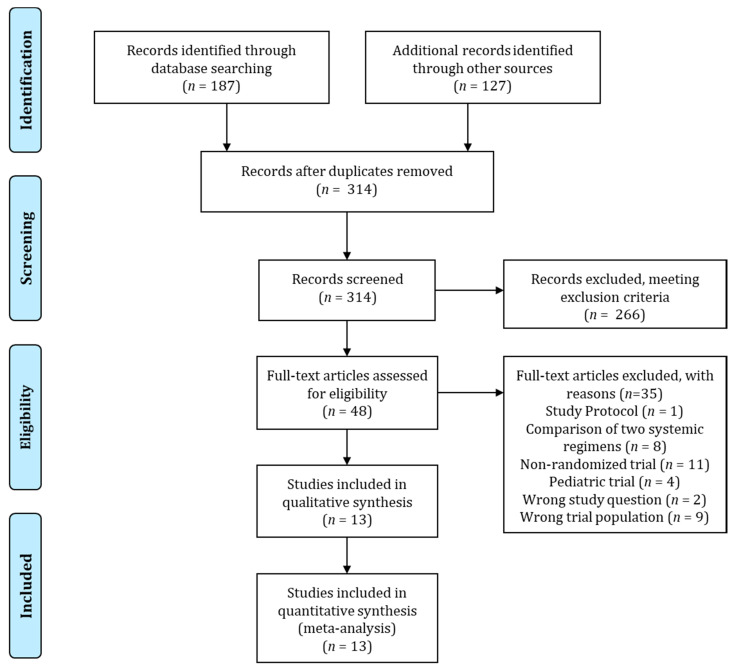
The PRISMA flow chart of the literature search.

The PRISMA flow chart for the literature search is presented in Figure 1.

**Figure 2:**
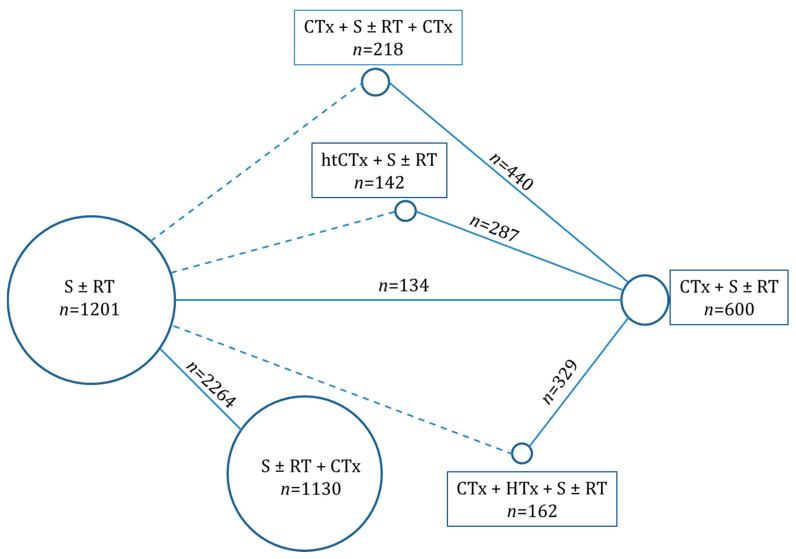
Overview of the network meta-analysis. Shown are the different treatment modalities with the direct comparisons (solid lines) and indirect comparisons (dashed lines) and the respective number of patients in the direct comparisons. Abbreviations: S = surgery, RT = radiotherapy, CTx = chemotherapy, HTx = hyperthermia, and htCTx = histology tailored chemotherapy.

Overall, we identified 13 publications that reported the results of 25 trials (*n* total = 3.465) which we analyzed in this network analysis. Twenty trials evaluated the effect of adjuvant chemotherapy in addition to local therapy alone. One trial compared neoadjuvant chemotherapy to surgery and/or radiotherapy [18], while the effect of perioperative chemotherapy in relation to neoadjuvant chemotherapy was analyzed in two trials [19,20]. Another study assessed the outcomes of neoadjuvant chemotherapy with or without additional regional hyperthermia [21,22]. Finally, one trial studied histology-tailored chemotherapy in relation to a standard chemotherapy regimen [23,24]. An overview of the network is presented in Figure 2 and a table of the included trials is presented in Table 1.

The adjusted network effect sizes of all indirect comparisons (perioperative chemotherapy, neoadjuvant chemotherapy in combination with regional hyperthermia, and tailored neoadjuvant chemotherapy) depended on one relatively small trial (*n* = 134) comparing neoadjuvant chemotherapy to immediate surgery ± radiotherapy [18]. However, the tests for inconsistency demonstrated no evidence for any endpoint of a differential estimate for direct or indirect comparison in the meta-analysis.

**Figure 3:**
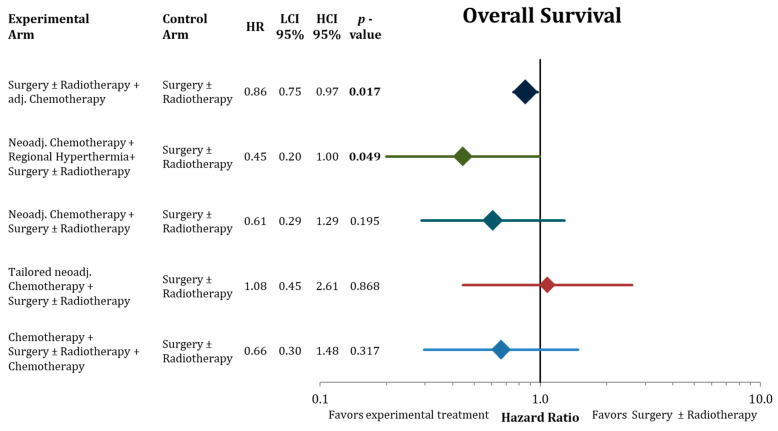
Forest plot comparing overall survival in the network analysis of the experimental treatments against surgery ± radiation therapy. Hazard ratios with the 95% confidence intervals and the corresponding *p*-values are presented. The size of the diamonds are proportional to the weights in the meta-analysis. HR = hazard ratio, LCI = lower limit of 95% confidence interval, and HCI = higher limit of 95% confidence interval. Bold values signify statistically significant values.

The network analysis regarding overall survival is shown in Figure 3. Compared to standard therapy of surgery ± radiotherapy, additional adjuvant chemotherapy (direct comparison, 20 trials) significantly improved overall survival (HR = 0.86; CI-95%: 0.75–0.97; *p* = 0.017). Neoadjuvant chemotherapy (direct comparison, 1 trial) and perioperative chemotherapy (indirect comparison) had higher effect sizes than adjuvant chemotherapy, but did not reach statistical significance ((HR = 0.61, CI-95%: 0.29–1.29, *p* = 0.195) and (HR = 0.66; CI-95%: 0.30–1.48; *p* = 0.317)). Tailored neoadjuvant chemotherapy (indirect comparison) had no statistical effect of significance (HR = 1.08; CI-95%: 0.45–2.61). Regional hyperthermia in combination with neoadjuvant chemotherapy (indirect comparison) resulted in the highest effect size of all tested modalities in the network (HR = 0.45; CI-95%: 0.20–1.00; *p* = 0.049).

**Figure 4:**
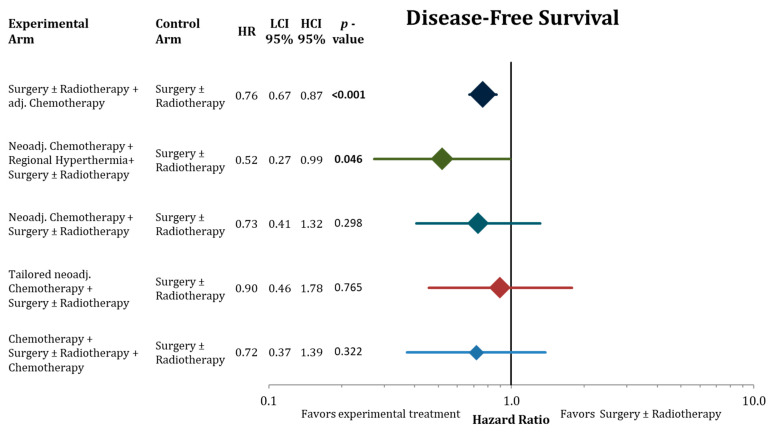
Forest plot comparing disease-free survival in the network analysis of the experimental treatments against surgery ± radiation therapy. Hazard ratios with 95% confidence intervals and the corresponding *p*-values are presented. The sizes of the diamonds are proportional to the weights in the meta-analysis. HR = hazard ratio, LCI = lower limit of 95% confidence interval, and HCI = higher limit of 95% confidence interval. Bold values indicate significant *p*-values.

**Figure 5:**
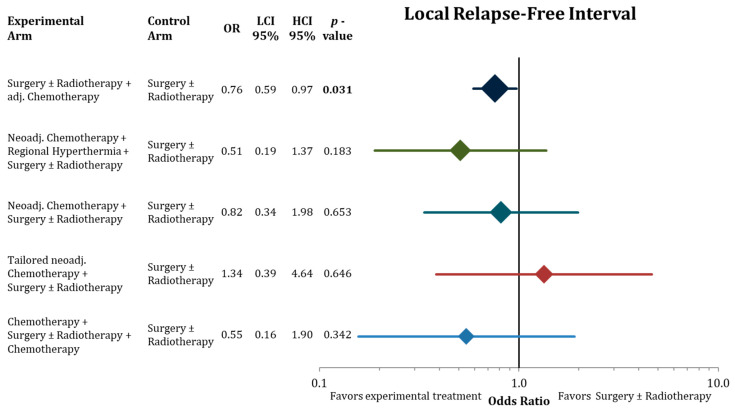
Forest plot comparing local relapse-free interval in the network analysis of the experimental treatments against surgery ± radiation therapy. Hazard ratios with lower and upper 95% confidence intervals and the corresponding *p*-values are presented. The sizes of the diamonds are proportional to the weights in the meta-analysis. HR = hazard ratio, LCI = low limit of 95% confidence interval, and HCI = high limit of 95% confidence interval. Bold values indicate significant *p*-values.

**Figure 6:**
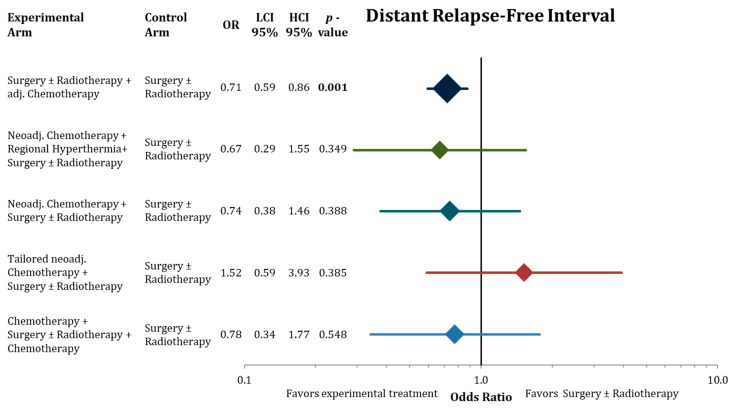
Forest plot comparing distant relapse-free interval in the network analysis of the experimental treatments against surgery ± radiation therapy. Hazard ratios with lower and higher 95% confidence interval and the corresponding *p*-values are presented. The sizes of the diamonds are proportional to the weights in the meta-analysis. HR = hazard ratio, LCI = lower limit of 95% confidence interval, and HCI = higher limit of 95% confidence interval. Bold values indicate significant *p*-values.

Figure 4, Figure 5 and Figure 6 illustrate the results of the network meta-analysis for the endpoints disease-free survival, local relapse-free interval, and distant relapse-free interval, respectively.

Adjuvant chemotherapy significantly improved DFS, LRFI, and DRFI compared to surgery ± radiotherapy. Neoadjuvant chemotherapy added to regional hyperthermia also resulted in significant longer DFS. The point estimates in the network with the corresponding confidence intervals for the residual treatment modalities did not reach significantly. However, we detected the same trends for the treatment modalities as in the analysis of OS, trials with neoadjuvant chemotherapy reporting favorable effect size estimates.

**Figure 7:**
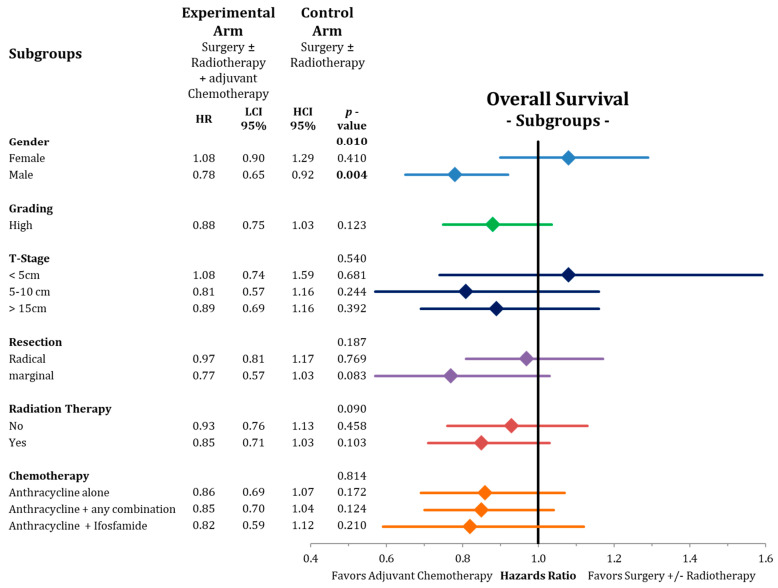
Forest plot showing the subgroup analysis for adjuvant chemotherapy for the endpoint of overall survival. Presented are hazard ratios with 95% confidence intervals with the corresponding *p*-values and the interaction tests. The size of the diamonds are proportional to the weights in the meta-analysis. HR = hazard ratio, LCI = lower limit of 95% confidence interval, and HCI = higher limit of 95% confidence interval. Bold values indicate significant *p*-values.

The subgroup analysis of overall survival depicted in Figure 7 showed a significant benefit for male patients (HR = 0.78; CI-95%: 0.65–0.92; *p* = 0.004) compared to female patients (HR = 1.08; CI-95%: 0.90–1.29; *p* = 0.410) receiving adjuvant chemotherapy with a significant test for heterogeneity (*p* = 0.010). We further detected a trend for longer overall survival for the marginally resected patients (HR = 0.77; CI-95%: 0.57–1.03; *p* = 0.083) with adjuvant chemotherapy. No differences were found for the other subgroups studied, such as T-stage, grading, radiotherapy, or type of chemotherapy (see Figure 7).

## 4. Discussion

We present the results of a network meta-analysis on the controversial topic of peri-operative chemotherapy in soft-tissue sarcoma. Surgery with risk-based additional radiotherapy is generally accepted as standard treatment for localized soft-tissue sarcomas of the extremities and trunk. The results of this standard treatment are favorable in low grade soft-tissue sarcomas but are unsatisfactory especially in large (>5 cm) high-grade soft-tissue sarcomas. The addition of systemic chemotherapy to the standard treatment in different sequences have been tested in several randomized trials with partially inconsistent results and several missing direct comparisons between treatment strategies. Figure 2 summarizes the tested treatment strategies and the available direct comparisons as well as the missing direct comparisons. The latter clinically relevant comparisons were indirectly estimated by means of network meta-analysis.

Adjuvant chemotherapy and neoadjuvant chemotherapy in combination with regional hyperthermia were the only treatment strategies in the network meta-analysis that lead to statistically longer overall survival in comparison to surgery with or without radiotherapy (Figure 4). When ranking the treatment strategies by effect sizes, a neoadjuvant chemotherapy component resulted in favorable effect sizes compared to adjuvant chemotherapy without reaching statistical significance compared to S ± RT. However, the considerably larger effect sizes of neoadjuvant chemotherapy in combination with hyperthermia (OR 0.45) compared to adjuvant chemotherapy (OR 0.86) indicates that this combined treatment might be a valid treatment option. Yet, access to regional hyperthermia is limited to specialized centers, and clinical evidence is based on the results of one single randomized trial that tested the addition of hyperthermia to naCTx alone [21]. Hence, the comparison to the control treatment (surgery ± radiotherapy) in the network meta-analysis was indirect. In addition, one needs to consider the main limitation of all three indirect comparisons in this network meta-analysis (Figure 7), which depended all on the effect size of one relatively small trial (*n* = 134) comparing neoadjuvant chemotherapy followed by surgery ± radiotherapy to immediate surgery ± radiotherapy [18]. Furthermore, in contrast to most other trials in the network, more than 50% of the patients in the hyperthermia trial had non-extremity sarcomas including many retroperitoneal sarcomas. The hyperthermia trial has also been criticized for employing etoposide besides doxorubicin and ifosfamide in the chemotherapy regimen in both arms of the trial. Although etoposide is known to be effective for soft-tissue sarcomas in pediatric protocols, its value for most adult-type soft-tissue sarcomas has been inadequately evaluated, potentially adding unnecessary toxicity. Accordingly, most treatment guidelines regard neoadjuvant chemotherapy in combination with hyperthermia as one possible treatment option among others for locally advanced high-grade soft-tissue sarcomas [48,49].

For sole neoadjuvant chemotherapy and perioperative chemotherapy (before and after surgery) the same before mentioned limitation account regarding the estimates in the network meta-analysis. Nevertheless, the effect size indicate that these treatments could be beneficial for high risk soft-tissue sarcoma patients (Figure 3, Figure 4, Figure 5 and Figure 6) compared to surgery ± radiotherapy and adjuvant chemotherapy. The number of patients in these trials is not sufficient to allow for reliable estimates of a possible advantage.

Disappointingly, histologically tailored neoadjuvant chemotherapy using trabectedin for high-grade myxoid liposarcoma, gemcitabine plus dacarbazine for leiomyosarcoma, high-dose ifosfamide for synovial sarcoma, etoposide plus ifosfamide for malignant peripheral nerve sheath tumors, and gemcitabine plus docetaxel for undifferentiated pleomorphic sarcoma, did not show a benefit in any investigated endpoint in the indirect comparison to surgery ± adjuvant radiotherapy (Figure 3, Figure 4, Figure 5 and Figure 6). In the direct comparison in the respective randomized trial, histotype-tailored neoadjuvant chemotherapy resulted in a significantly worse overall survival than neoadjuvant epirubicin plus ifosfamide irrespective of the histological subtype [23,24].

In this analysis, we present an update on the benefit of adjuvant chemotherapy in adult soft-tissue sarcoma (20 trials, 2264 patients) beyond the previous assessments [7,17]. Adding postoperative chemotherapy to surgery ± radiotherapy results in a moderate, but statistically significant, advantage in all important oncological outcomes including overall survival (Figure 4, Figure 5 and Figure 6). Compared to the publication of the “Sarcoma Meta-Analysis Collaboration” (SMAC) that reported a significant benefit for adjuvant chemotherapy for local, distant, and overall relapse free survival, but not for overall survival [6], in the current meta-analysis the results of seven additional randomized trial with a total of an additional 824 patients were available. In contrast to the SMAC, the current meta-analysis was not based on individual patient’s data, however, for the older trials (63% of patients) we used the published hazard ratios from the SMAC based on the individual patient’s data. No significant heterogeneity was found between the old trials in the SMAC and the new trials. In terms of overall survival, the estimated OR of 0.86 (CI-95%: 0.75–0.97) indicates an absolute difference at 10 years of 5.3% (CI-95%: 1.1–9.7%). In view of this relatively small advantage, we performed a subgroup analysis for adjuvant chemotherapy in the network meta-analysis to identify patients, who may have larger benefits from adjuvant chemotherapy. Besides some non-significant trends indicating larger benefits in case of marginal resection and for patients, who had received radiation therapy, the most relevant finding was the substantial survival advantage after adjuvant chemotherapy in male patients (8.5% absolute at 10 years) and the lack of any benefit in female patients (Figure 7). The same observation with similar effect sizes for both genders were already reported in the publication of the SMAC [17] without any commentary in the discussion, and in a pooled analysis of two EORTC trials [50]. Importantly, we were unable to perform a multivariate analysis based on the available published. Moreover, no multivariate analyses are available from the SMAC or the pooled analysis of the two EORTC trials, leaving the question of gender being an independent predictive factor for the benefit from chemotherapy unanswered [6]. When excluding the uterine sarcomas from the analysis, we did not observe any changes to the outcomes of this analysis Hormone receptors (estrogen and androgen) have been found preferentially on liposarcomas, but not on other histological subtypes [51]. Thus, the influence of gender on the efficacy of adjuvant chemotherapy needs further prospective evaluation.

Systemic therapy in soft-tissue sarcomas remains a grey area where additional factors like localization of the tumor, histological subtype, feasibility of the surgical resection, type, and dosing of the systemic therapy should be considered when making a treatment decision. Further, the data analyses might be limited by the small sample size and the fact that patients from randomized trials might not reflect the real world patient population. However, complementary to the data from the randomized studies, Graham et al. used the National Cancer Database (NCDB) to observe the association between overall survival and chemotherapy in 5436 patients with the five most common subtypes of soft-tissue sarcoma with primary disease localized to the extremity or trunk [52]. Multi-agent chemotherapy was associated with improved overall survival in numerous subgroups, including patients with larger tumors (>5 cm), those treated at high-volume medical centers, or those who received radiation therapy. They also found an overall survival benefit to multi-agent chemotherapy amongst the elderly (>70 years) and African American patients. Neither younger age nor chemotherapy timing was associated with better clinical outcomes [52]. Overall, these supplementing real-world data match the results from our analysis.

Numerous attempts have been published predicting the individual patient’s prognosis using validated nomograms [53,54]. Pasquali and colleagues were able to predict the benefit from adjuvant chemotherapy from the randomized EORTC-STBSG 62931 trial grouping the study population into three subgroups according to the prognostic nomogram Sarculator. Another approach that might be incorporated in future treatment paradigms is the use of gene expression signatures like CINSARC [55]. A potential advantage of this method would be the applicability beyond distinct histological subtypes.

## 5. Conclusions

The comparison of different treatment strategies for the treatment of adult soft-tissue sarcoma in a network meta-analysis demonstrates that adjuvant chemotherapy and neoadjvuant chemotherapy combined the regional hyperthermia improve overall survival. The modest benefit of adjuvant chemotherapy might be restricted to high-risk patients and male gender. Neoadjuvant chemotherapy strategies might be more effective than adjuvant therapy, but the conclusion are limited by the number of included patients and available randomized studies.

This work was presented at the Annual ASCO Meeting in 2020.

## Figures and Tables

**Table 1:** Overview of included trials. * = baseline characteristics from SMAC meta-analysis.

Trial	Year	N Total	Med. AGE	Female	FU (y)	Site	G3+	Exp. Arm	*n*	Contr. Arm	*n*	Surgery	RT	CTx
GOG (Omura) [25]	1997	225	n.a.	100%	9.4 *	U	67% *	adCTx	113	S ± RT	112	HE	n.a.	D
MDA (Benjamin) [6,26]	1997	54	n.a.	54% *	9.4 *	Extr.,T	67% *	adCTx	26	S ± RT	28	n.a.	n.a.	D + C + ACTD + V
Mayo (Edmonson) [27,28]	1997	57	n.a.	54% *	9.4 *	Extr., T	67% *	adCTx	28	S ± RT	29	n.a	n.a.	D + V + ACTD + DTIC
NCI4 (Rosenberg/Chang) [29,30]	1997	25	n.a.	54% *	9.4 *	Extr.	67% *	adCTx	17	S ± RT	8	Amp or WE	45–70 Gy	D + C + MTX
NCI5 (Glenn) [31,32]	1997	79	n.a.	54% *	9.4 *	T, HN, RP, B	67% *	adCTx	38	S ± RT	41	WE	60–63 Gy	D + C + MTX
NCI6 (Rosenberg/Chang) [29,30]	1997	41	n.a.	54% *	9.4 *	Extr.	67% *	adCTx	21	S ± RT	20	n.a.	n.a.	D + C + MTX
EORTC (Bramwell) [16]	1997	467	n.a.	54% *	9.4 *	Extr., T, HN	67% *	adCTx	234	S ± RT	233	n.a.	40–50 Gy	D + C + V + DTIC
DFCI/MGH (Antman) [33]	1997	46	n.a.	54% *	9.4 *	Extr. T, HN, RP	67% *	adCTx	21	S ± RT	25	Amp or WE	62.5–67.5 Gy	D
ECOG (Lerner) [34]	1997	47	n.a.	54% *	9.4 *	Extr., T, HN, RP	67% *	adCTx	24	S ± RT	23	Amp or WE	50–64 Gy	D
Bergonie (Ravaud) [35]	1997	65	n.a.	54% *	9.4 *	Extr., T, HN, RP	67% *	adCTx	33	S ± RT	32	n.a.	n.a.	D + C + V + DTIC
SSG (Alvegard) [36]	1997	240	57	49%	9.4 *	Extr., T, HN, B, Tx, Abd.	100%	adCTx	121	S ± RT	119	WE	42–51 Gy	D
Rizzoli (Gherlinzoni/Picci) [37,38]	1997	77	n.a.	54% *	9.4 *	Extr.	67% *	adCTx	34	S ± RT	43	WE	45 Gy	D
IGSC (Baker/Antman) [39,40]	1997	92	n.a.	54% *	9.4 *	Extr., T, HN, RP	67% *	adCTx	43	S ± RT	49	n.a.	n.a.	D
SAKK [6]	1997	29	n.a.	54% *	9.4 *	Extr., T	67% *	adCTx	14	S ± RT	15	n.a.	n.a.	D + IFO
Pautier [41]	2013	81	55	100%	4.3	U	n.a.	adCTx	39	S + RT	42	n.a.	45/1.8 Gy	D + IFO + CDDP
Woll [8]	2012	351	49	45%	8	Extr., T, HN	46%	adCTx	175	S ± RT	176	n.a.	50–66 Gy	D + IFO
Fakrai [42]	2010	58	52	46%	8.1	Extr., T, RP	73%	adCTx	31	S + RT	27	“adequate”	51/1.7 Gy	D + IFO + DTIC
Frustaci [43,44]	2001/2003	104	n.a.	41%	4.9	Extr., T	100%	adCTx	53	S ± RT	51	HE	44.8–66 Gy	EPI + IFO
Petrioli [45]	2002	88	53	49%	7.8	Extr., T, Abd., RP	41%	adCTx	45	S ± RT	43	WE	54–60 Gy	EPI + IFO
Hensley [46]	2018	38	n.a.	100%	4.3	U	100%	adCTx	20	S	18	HE	n.a.	TXT + GEM + D
Issels [21,22]	2018	341	52	44%	11.3	Extr., T, HN	53%	NaCTx+HTx	162	NaCTx	167	n.a	50–66 Gy	D + IFO
Gronchi [23,24]	2017/-20	286	40	38%	4.3	Extr., T, Abd.	100%	HtCTx	142	NaCTx	144	n.a	44–66 Gy	EPI + IFO
Gortzak [18]	2001	134	53	40%	7.3	Extr., T, HN	n.a.	NacCTx	67	S ± RT	67	Amp or WE	60–80 Gy	D + IFO
Gronchi [20,47]	2016	321	49	n.a.	9.75	Extr., T	n.a.	NaCTx	161	periCTx	160	n.a	44–66 Gy	EPI + IFO
Eilber [19]	1988	119	59	45%	2.3	Extr.	100%	periCTx	57	RT + S	62	Amp or WE	17.5/3.5 Gy	D

Abbreviations: n.a. = not available, adCTx = adjuvant chemotherapy, NaCTx + HTx = neoadjuvant chemotherapy and hyperthermia, NaCTx = neoadjuvant chemotherapy, HtCTx = histology tailored chemotherapy, periCTx = perioperative chemotherapy, HE = hysterectomy, Amp = amputation, WE = wide excision, Gy = gray, G3+ = grade 3 or higher, FU = follow-up, D = Doxorubicine, C = Cyclosphosphamid, ACTD = Dactinomycin; V = Vincristine; DTIC = Dacarbazine; MTX = Methotrexate; IFO = Ifosfamide, CDDP = Cisplatin, GEM = gemcitabine, EPI = Epirubicin, TXT = docetaxel, T = Trunk, B = Breast; RP = Retroperitoneal, Abd = Abdominal; U = Uterus; HN = head and neck, and Tx = Thorax, y = year; Patients in the treated in the arms with systemic treatment were intended to receive local therapy as well.

## Abbreviations

Abd	abdominal
ACTD	dactinomycin
adCTx	adjuvant chemotherapy
Amp	amputation
B	breast
C	cyclophosphamide
CDDP	cisplatin
CI	confidence interval
CTx	chemotherapy
D	doxorubicin
DFS	disease-free survival
DRFI	distant recurrence-free interval
DTIC	dacarbazine
EORTC	European Organization for Research and Treatment of Cancer
EPI	epirubicin
FU	follow-up
G	grade
GEM	gemcitabine
GIST	gastrointestinal stromal tumors
Gy	gray
HCI	high limit of 95% confidence interval
HE	hysterectomy
HN	head and neck
HTx	hyperthermia
HtCTx	histology tailored chemotherapy
IFO	ifosfamide
ivhet	inverse-variance heterogeneity
LCI	low limit of 95% confidence interval
LRFI	local recurrence-free interval
MTX	methotrexate
n.a.	not available
NCDB	National Cancer Database
n.r.	not reported
NaCTx + HTx	neoadjuvant chemotherapy and hyperthermia
NaCTx	neoadjuvant chemotherapy
OR	odds ratio
OS	overall survival
periCTx	perioperative chemotherapy
R	resection rate
RP	retroperitoneal
RT	radiotherapy
S	surgery
T	trunk
Tx	thorax
TXT	docetaxel
U	uterus
V	vincristine
WE	wide excision
Y	year
